# High Catalytic Selectivity of Electron/Proton Dual‐Conductive Sulfonated Polyaniline Micropore Encased IrO_2_ Electrocatalyst by Screening Effect for Oxygen Evolution of Seawater Electrolysis

**DOI:** 10.1002/advs.202412862

**Published:** 2024-12-04

**Authors:** Yuhan Shen, Shengqiu Zhao, Fanglin Wu, Hao Zhang, Liyan Zhu, Mingjuan Wu, Tian Tian, Haolin Tang

**Affiliations:** ^1^ State Key Laboratory of Advanced Technology for Materials Synthesis and Processing Wuhan University of Technology Wuhan 430070 P. R. China; ^2^ National Energy Key Laboratory for New Hydrogen‐Ammonia Energy Technologies Foshan Xianhu Laboratory Foshan 528200 P. R. China; ^3^ Hubei Key Laboratory of Fuel Cell Wuhan 430070 P. R. China

**Keywords:** catalytic selectivity, conjugate organic framework, IrO_2_, seawater electrolysis, sulfonate

## Abstract

Acidic seawater electrolysis offers significant advantages in high efficiency and sustainable hydrogen production. However, in situ electrolysis of acidic seawater remains a challenge. Herein, a stable and efficient catalyst (SPTTPAB/IrO_2_) is developed by coating iridium oxide (IrO_2_) with a microporous conjugated organic framework functionalized with sulfonate groups (‐SO_3_H) to tackle these challenges. The SPTTPAB/IrO_2_ presents a ‐SO_3_H concentration of 5.62 × 10^−4^ mol g^−1^ and micropore below 2 nm numbering 1.026 × 10^16^ g^−1^. Molecular dynamics simulations demonstrate that the conjugated organic framework blocked 98.62% of Cl^−^ in seawater from reaching the catalyst. This structure combines electron conductivity from the organic framework and proton conductivity from ‐SO_3_H, weakens the Cl^−^ adsorption, and suppresses metal‐chlorine coupling, thus enhancing the catalytic activity and selectivity. As a result, the overpotential for the oxygen evolution reaction (OER) is only 283 mV@10 mA cm^−2^, with a Tafel slope of 16.33 mV dec^−1^, which reduces 13.8% and 37.8% compared to commercial IrO_2_, respectively. Impressively, SPTTPAB/IrO_2_ exhibits outstanding seawater electrolysis performance, with a 35.3% improvement over IrO_2_ to 69 mA cm^−2^@1.9 V, while the degradation rate (0.018 mA h^−1^) is only 24.6% of IrO_2_. This study offers an innovative solution for designing high‐performance seawater electrolysis electrocatalysts.

## Graphical abstract

A stable and efficient catalyst (SPTTPAB/IrO_2_) is developed by coating iridium oxide (IrO_2_) with sulfonate groups (‐SO_3_H) modified microporous conjugated organic framework. This proton/electron dual‐conducting microporous conjugated organic framework effectively filters the substances reaching the surface of IrO_2_, ultimately achieving efficient and stable in‐situ electrolysis in acidic seawater conditions.

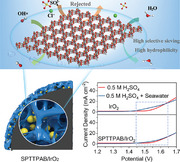

## 1 Introduction

Hydrogen production by water electrolysis combined with renewable energy sources (such as solar, wind, water, etc.) is widely considered a much superior strategy to effectively mitigate the environmental problems caused by existing fossil energy sources.^[^ ^1^, ^2^, ^3^, ^4^ ^]^ Proton exchange membrane water electrolyzer (PEMWE) is regarded as one of the most promising technologies for large‐scale hydrogen production using renewable energy and clean power due to its advantages of low gas crossover, high hydrogen production efficiency, and fast response to load changes.^[^ ^5^ ^]^ However, the primary catalyst used in this technology is the precious metal oxide represented by iridium oxide (IrO_2_), which has weak catalytic selectivity for ions and struggles to operate under high potential and corrosive environments. As a result, its application is limited to pure water electrolysis and proves ineffective in more complex systems, particularly in seawater electrolysis. This weak catalytic selectivity and stability of IrO_2_ have seriously hindered the development of PEMWE.^[^ ^6^ ^]^ Therefore, there is an urgent need to develop a catalyst with enhanced durability and ion selectivity.^[^ ^7^, ^8^, ^9^ ^]^

Although most of the rutile structure catalysts represented by IrO_2_ can show certain performance in seawater electrolysis, its activity and stability are far beneath the demand for industrialization.^[^ ^10^ ^]^ In addition, catalysts with special morphologies (such as hollow structures and hierarchical porous structures) have demonstrated significant advantages in electrocatalytic reactions, enhancing catalytic activity and stability.^[^ ^11^, ^12^, ^13^, ^14^, ^15^, ^16^ ^]^ However, due to the lower activation barrier of the chlorine evolution reaction (CER) compared to the oxygen evolution reaction (OER), side reactions still occur simultaneously during seawater electrolysis.^[^ ^17^, ^18^ ^]^ Therefore, an intermediate layer is often prepared on the catalyst surface to enhance catalytic selectivity in seawater and ensure optimal performance.^[^ ^19^ ^]^ For example, Karthikeyan et al. prepared a novel seawater electrolysis catalyst IrO_2_@MnO_2_/rGO by filling IrO_2_ particles in a multilayer structure of MnO_2_ and graphene oxide, with an overpotential of only 190 mV@10 mA cm^−2^ in alkaline seawater.^[^ ^20^ ^]^ Gan et al. loaded IrO_x_ with BaCO_3_ as the carrier to prepare a catalyst that can slowly electrolyze water to produce hydrogen in alkaline seawater, which has good selectivity and corrosion resistance.^[^ ^21^ ^]^ Haik et al. developed a nano‐micro heterostructure with NiOOH nanosheets embedded in a Ni(OH)_2_ microarray, functionalized with SO_x_ groups to form a cation‐selective protective layer.^[^ ^22^ ^]^ This layer impedes Cl^−^ ions diffusion and abstracts hydrogen from reaction intermediates, significantly enhancing the selectivity and corrosion resistance of the catalyst. The catalyst achieves a current density of 1 A cm^−^^2^ at a low input voltage of 400 mV and operates in unpurified seawater for over 168 h without degradation or hypochlorite formation. However, most catalyst interlayer designs are limited to alkaline seawater electrolysis, while research on acidic and neutral seawater electrolysis remains scarce. In addition, due to Oswald curing and the instability of transition metals in lye, catalysts with such composite structures are prone to phase separation or the formation of metal oxides with poor conductivity.^[^ ^23^ ^]^

In this work, we propose constructing a porous conjugated organic framework on the catalyst surface to eliminate the challenges catalysts face in seawater electrolysis. A stable and efficient catalyst (SPTTPAB/IrO_2_) was obtained by constructing a sulfonated conjugated microporous polymer poly[1,3,5‐tris(4‐diphenylaminophenyl)benzene] (SPTTPAB) on the surface of IrO_2_ by coupling and chemical deposition methods. The porous polymer structure on the catalyst surface combines the electronic conductivity of the conjugated organic framework and the proton conductivity of the sulfonate groups, which promotes the transport of electrons and protons in the system, while selectively screening the substances reaching the catalyst surface and restricting the participation of the Cl^−^ ions in the reaction, thus improving the catalytic selectivity and activity.^[^ ^24^, ^25^, ^26^ ^]^ As a result, SPTTPAB/IrO_2_ overcame the low efficiency and low durability of OER reaction catalysts in seawater electrolysis, which is verified through experiments and molecular dynamics (MD) simulations. This work addresses key challenges in seawater electrolysis by mitigating side reactions such as chlorine evolution, significantly improving the catalytic performance of IrO_2,_ and offering a more sustainable and efficient pathway for seawater electrolysis, with potential applications at both laboratory and industrial scales. Looking forward, these principles may be extended to other complex electrochemical systems, including desalination and neutral seawater electrolysis, further advancing the development of electrocatalysts for large‐scale clean hydrogen production and contributing to a low‐carbon energy future.

## 2 Results and Discussion

### 2.1 Structural Characterization of Catalysts

**Figure 1:**
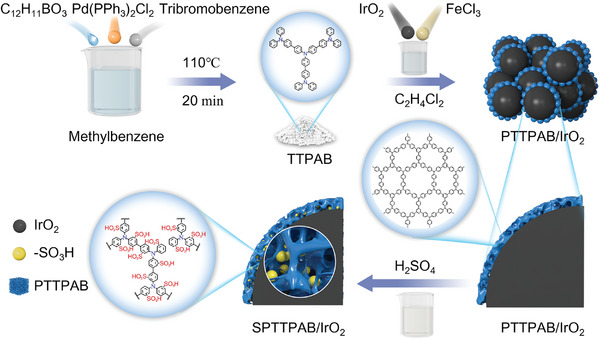
Schematic of the synthetic route of SPTTPAB/IrO_2_.

**Figure 2:**
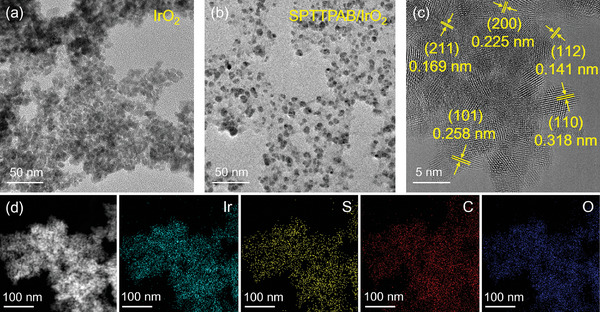
TEM image of a) IrO_2_, b) SPTTPAB/IrO_2_. c) HRTEM image, d) relevant elemental mapping images of SPTTPAB/IrO_2_.

The schematic synthesis pathway of the SPTTPAB/IrO_2_ core‐shell catalyst is presented in **Figure** **1**. Firstly, TTPAB was synthesized by the Suzuki coupling method, then TTPAB was deposited on the surface of IrO_2_ crystals by oxidative cross‐linking method and finally sulfonated to obtain the final product SPTTPAB/IrO_2_. Comparing the TEM images of IrO_2_ and SPTTPAB/IrO_2_, it can be identified that SPTTPAB/IrO_2_ is composed of lamellar deposited SPTTPAB wrapped around granular IrO_2_ (**Figure** **2**). Among them, lattice stripes with spacings of 0.318 and 0.258 nm appeared in the high‐resolution TEM images of SPTTPAB/IrO_2_, which matched the (110) and (101) crystal faces of rutile IrO_2_, respectively (Figure 2c). Interestingly, the average particle size of IrO_2_ decreased from 219 to 87 nm (Figure S3a, Supporting Information), and the zeta potential decreased from −14.96 to −27.97 mV (Figure S3b, Supporting Information) after being coated by 5 wt.% SPTTPAB, indicating that the better dispersion of SPTTA/IrO_2_ compared to IrO_2_ (corresponding to the results of Figure 2a,b). In addition, the EDS mapping image shows that the IrO_2_ nanoparticles are uniformly surrounded by SPTTPAB (Figure 2d), indicating that the IrO_2_ catalyst is homogeneously distributed in SPTTPAB and does not generate large agglomeration, which facilitates the exposure of more active sites. Moreover, the adverse effect of sulfonation on the electronic conductivity of the conjugated polymer PTTPAB is negligible, resulting in a 1.7‐fold increase in the electronic conductivity of SPTTPAB/IrO_2_ compared to IrO_2_ (Figure S3c, Supporting Information).

**Figure 3:**
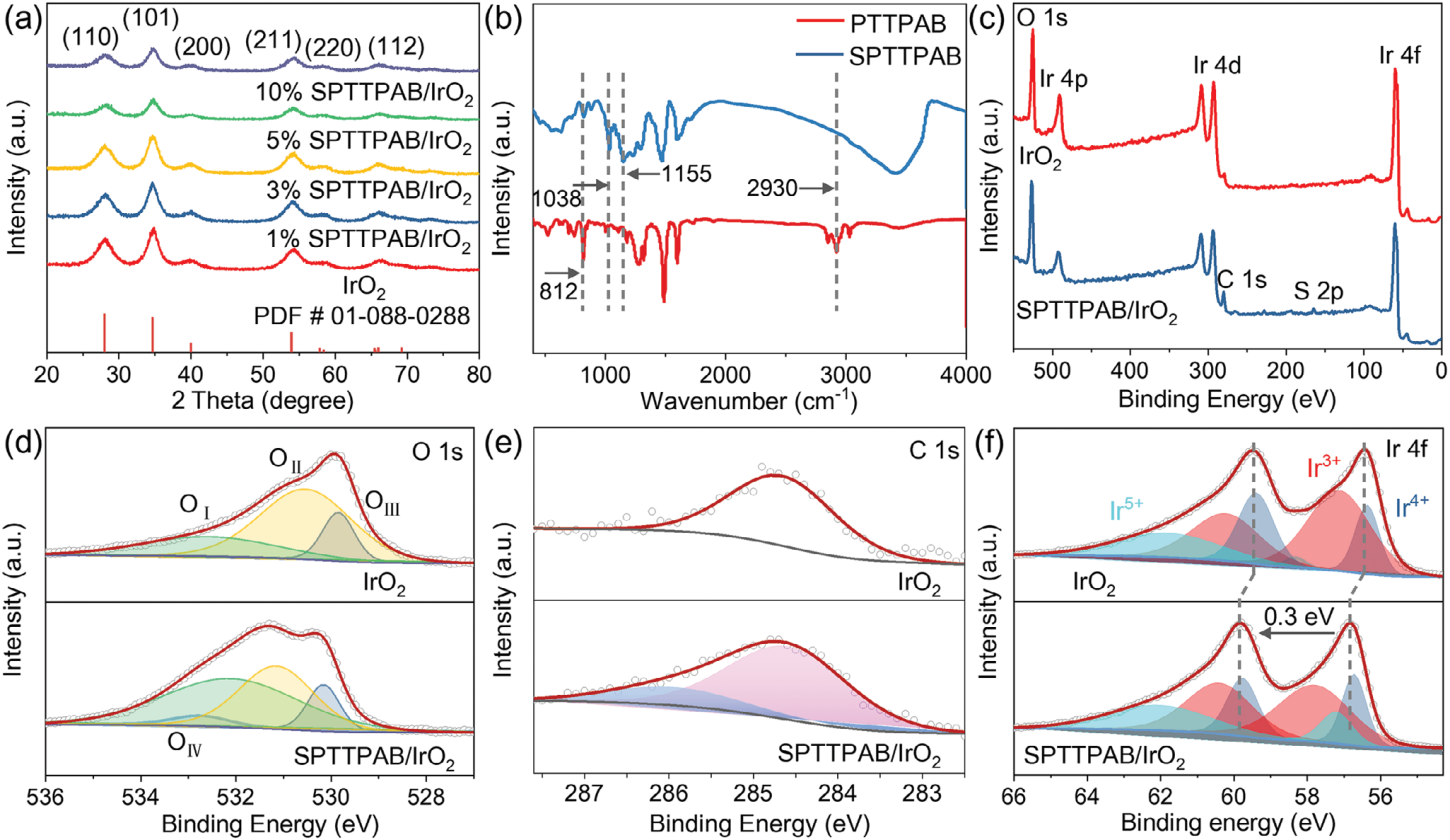
Characterization of the physicochemical structure of IrO_2_ and SPTTPAB/IrO_2_. a) XRD patterns, b) FTIR spectrum, c) XPS full spectra. XPS spectra of d) O 1s, e) C 1s, and f) Ir 4f.

The XRD patterns in **Figure** **3**a unveil all the diffraction peaks that correspond well to the rutile phase of IrO_2_ (PDF#01‐088‐0288), indicating that the sulfonation step has no significant effect on the crystal phase of IrO_2_. The FT‐IR is employed to verify the successful grafting of sulfonate groups on PTTPAB (Figure 3b). The peaks at 2930 and 812 cm^−1^ belong to the antisymmetric stretching of C─H on the branch chain and the out‐of‐plane bending of ═C─H on the benzene ring, respectively.^[^ ^27^ ^]^ The intensity of both peaks is significantly weakened after the grafting reaction, suggesting that the functional groups at these positions have been replaced by ─SO_3_H.^[^ ^28^ ^]^ Concurrently, peaks at 1038 and 1155 cm^−1^, corresponding to the symmetric and antisymmetric expansion of aromatic sulfonate groups, validate the stable existence of sulfonate groups in SPTTPAB.^[^ ^29^ ^]^ The XPS was employed to delve more deeply into the chemical composition of these catalysts. As illustrated in Figure 3c, the XPS full spectra reveal the presence of carbon, sulfur, oxygen, and iridium within the catalysts. As displayed in Figure 3d, O_I_, O_II,_ and O_III_ at ≈532.5, ≈530.8, and ≈529.8 eV in the O 1s spectra are ascribed to the oxygen in the surface absorbed hydroxyl groups, oxygen vacancies and metal‐oxygen bond.^[^ ^11^, ^12^ ^]^ In addition, the peak at 532.8 eV observed is attributed to the S═O bond in ‐SO_3_H of SPTTPAB. Compared with pure IrO_2_, the O1s adsorption peaks of SPTTPAB/IrO_2_ were significantly enhanced, which was mainly attributed to the fact that the coating of SPTTPAB increased the hydrophilicity of the catalyst and thus absorbed more hydroxyl groups on the surface. According to the mass distribution of elements (Table S1, Supporting Information), the amount of sulfonate groups in the 5% SPTTPAB/IrO_2_ can be calculated as 5.62 × 10^−4^ mol g^−1^. The peak at 286 eV in the C 1s spectra corresponds to the π bond of the benzene ring, mainly derived from the PTTPAB conjugated polymer backbone (Figure 3e). We next perform Ir 4f spectra for IrO_2_ and SPTTPAB/IrO_2_. In Figure 3f, the Ir 4f spectra of the catalyst show multi‐peak characteristics. After sulfonate, the spectra of Ir 4f showed a positive shift of 0.3 eV, indicating that the electronic structure of the Ir site had changed. The peaks at 57.3 and 60.3 eV belong to 4f_7/2_ and 4f_5/2_ of Ir^3+^, respectively, and the peaks at 56.5 and 59.5 eV belong to 4f_7/2_ and 4f_5/2_ of Ir^4+^, respectively.^[^ ^30^, ^31^, ^32^, ^33^ ^]^ The peak area percentages of Ir^5+^ are higher in SPTTPAB/IrO_2_ than in IrO_2_ because the iridium oxide is in a higher energy state after being oxidized by sulfuric acid, which is more conducive to the catalytic reaction.^[^ ^34^, ^35^ ^]^

### 2.2 Electrochemical Properties of SPTTPAB/IrO_2_ in Seawater

**Figure 4:**
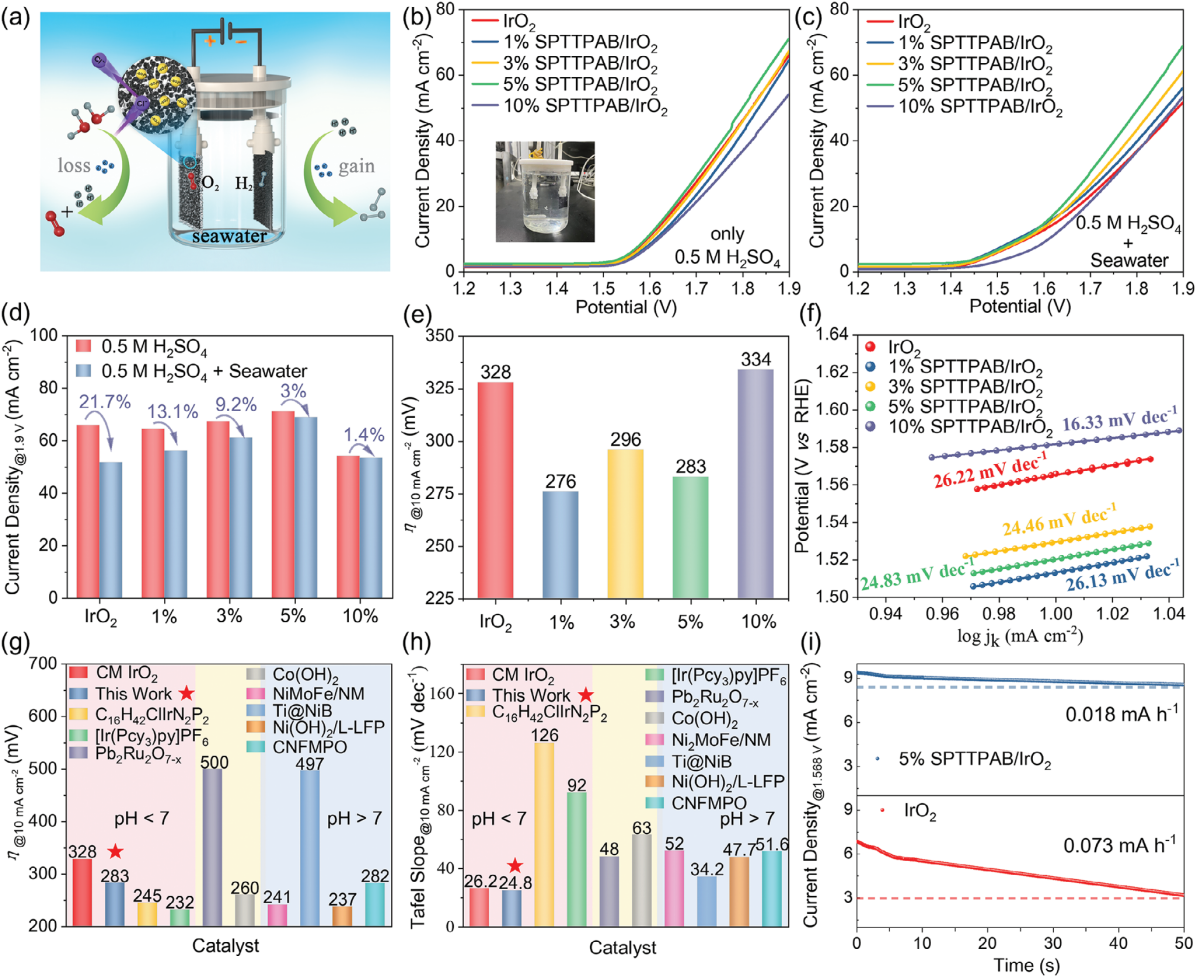
a) Schematic diagram of the working principle of the water electrolysis device. LSV curves for integral water electrolysis using different electrolytes: b) 0.5 m H_2_SO_4_ solution, c) acid artificial seawater. d) Variation of current density at a voltage of 1.9 V in pure acidic electrolyte and acidic electrolyte containing artificial seawater. e) OER overpotential at 10 mA cm^−2^ and f) Tafel plots in acid artificial seawater electrolyte. Comparison of g) overpotential h) Tafel slope of different seawater electrolytic catalysts at 10 mA cm^−2^. i) Stability test of 5 wt.% SPTTPAB/IrO_2_|50 wt.% Pt/C and IrO_2_|50 wt.% Pt/C at constant voltage 1.568 V (25 °C, 0.5 m H_2_SO_4_, 0.1 m NaCl, N_2_ saturation).

In order to demonstrate the feasibility of the as‐prepared SPTTPAB/IrO_2_ in seawater electrolysis, an integrated electrolysis device was constructed in which 5 wt.% SPTTPAB/IrO_2_ or IrO_2_ were used as anode catalyst and Pt/C (50 wt.% Pt) as cathode catalyst (**Figure** **4**a). As shown in Figure 4b, only the catalytic activity of 5 wt% SPTTPAB/IrO_2_ was slightly higher than that of IrO_2_ in 0.5 m H_2_SO_4_ electrolyte. However, the 5 wt.% SPTTPAB/IrO_2_ has an electrolytic performance of 69 mA@1.9 V in acid seawater electrolyte, which is elevated by 35.3% compared to IrO_2_ (Figure 4c). A comparison of the electrolytic performance before and after the incorporation of seawater in the electrolyte shows that the current density attenuation decreases significantly as the proportion of SPTTPAB increases (Figure 4d). Moreover, the overpotential of 5 wt.% SPTTPAB/IrO_2_ in acid seawater electrolyte is only 283 mV at the current density of 10 mA cm^−2^ (Figure 4e), and the Tafel slope is as low as 24.83 mV dec^−1^, where the Tafel slope of the 10 wt.% SPTTPAB/IrO_2_ is even only 16.33 mV dec^−1^ (Figure 4f). The results show that SPTTPAB/IrO_2_ has excellent catalytic selectivity and anti‐interference ability. Indeed, the overpotential and Tafel slope of 5 wt.% SPTTPAB/IrO_2_ are superior to other catalysts recently reported in the literature, as shown in Figures 4g,h.^[^ ^3^, ^17^, ^36^, ^37^, ^38^, ^39^, ^40^ ^]^

Additionally, the stability of 5 wt.% SPTTPAB/IrO_2_|50 wt.% Pt/C and IrO_2_|50 wt.% Pt/C was verified by a 9‐h constant voltage electrolysis test, as shown in Figure 4i. The current density of 5 wt.% SPTTPAB/IrO_2_|50 wt.%Pt/C was consistently higher than that of IrO_2_|50 wt.%Pt/C throughout the stability test. After 50 h of constant voltage stability testing, the current density of 5 wt.% SPTTPAB/IrO_2_|50 wt.%Pt/C remained stable (only dropping from 9.4 to 8.5 mA cm^−2^), with a decay rate as low as 0.018 mA h^−1^, which is only 24.6% of that of IrO_2_, demonstrating the excellent stability of SPTTPAB/IrO_2_ in artificial seawater. Afterward, the morphology and structure of the SPTTPAB/IrO_2_ after durability testing were verified by TEM and XRD. It is clear from the TEM image and XRD pattern (Figure S4, Supporting Information) that the morphology and crystal structure of the SPTTPAB/IrO_2_ remains basically unchanged after the reaction, which reflects the good stability of the materials.

### 2.3 The Influencing Factors of OER Performance in Seawater and Mechanism Analysis of SPTTPAB/IrO_2_ Catalyst

**Figure 5:**
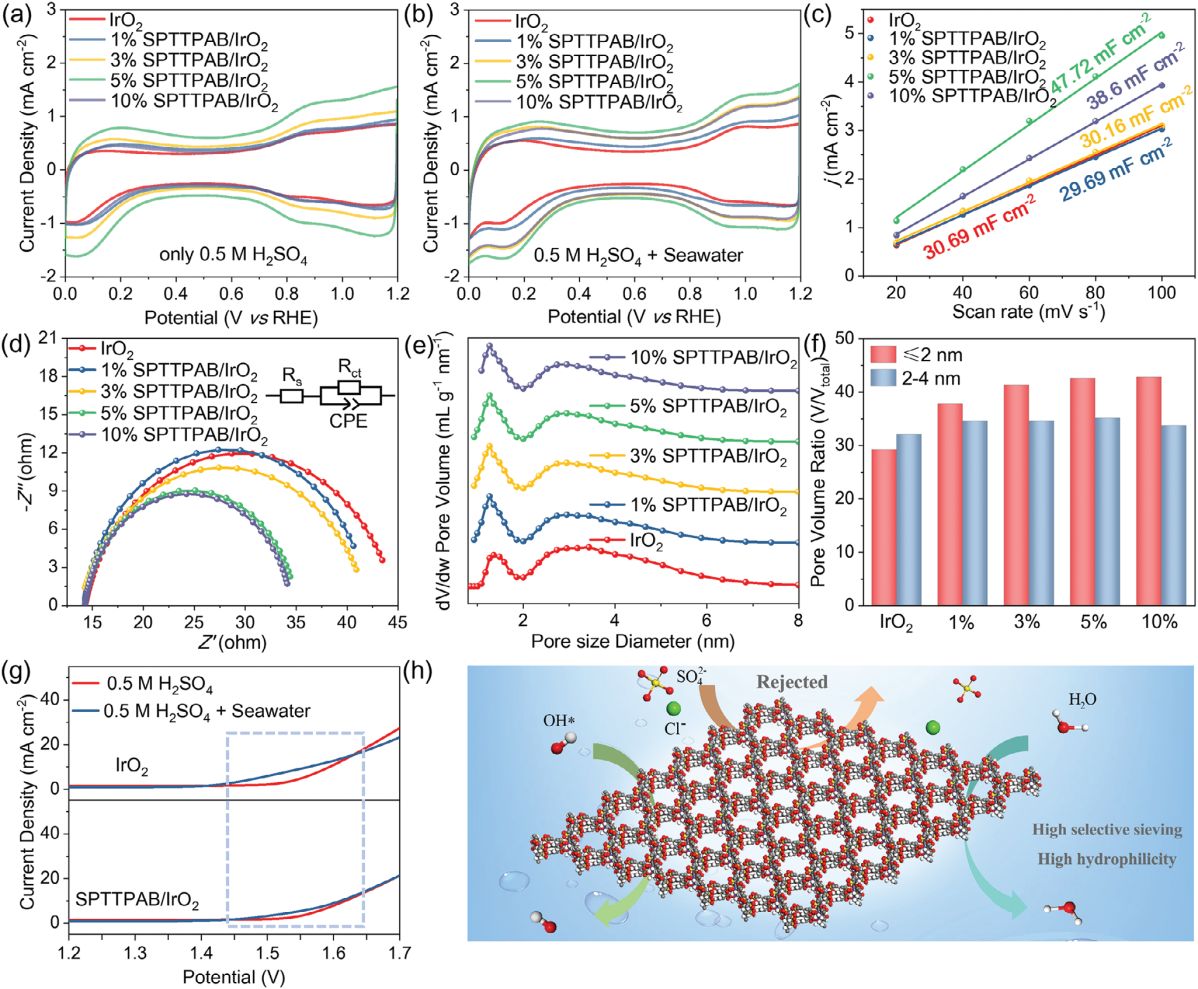
Cyclic voltammetry curves were recorded at a scanning rate of 20 mV s^−1^ in different electrolytes: a) 0.5 m H_2_SO_4_ solution, b) acid artificial seawater. c) The double capacitance, d) Nyquist plot of IrO_2_ and SPTTPAB/IrO_2_. The inset in Figure 5d is an equivalent fitted circuit diagram where Rs, Rct, and CPE denote ohmic resistance, charge transfer impedance, and constant phase element, respectively. e) Pore size distribution curves of IrO_2_ and SPTTPAB/IrO_2_ were measured using the BET method. f) The pore volume ratio of IrO_2_ and SPTTPAB/IrO_2_. g) Comparison of LSV curves of IrO_2_ and SPTTPAB/IrO_2_ in 0.5 m H_2_SO_4_ solution and acid artificial seawater. h) Mechanism diagram of SPTTPAB inhibiting side reactions in seawater electrolysis.

To elucidate the mechanism by which the SPTTPAB coating influences catalyst performance, electrochemical tests were conducted on IrO_2_ catalysts coated with different ratios of SPTTPAB in a three‐electrode system. The active site number of oxide catalysts used for electrochemical reactions, particularly in the OER, is usually related to the voltammetric charge (*q*), which can be quantified by integrating cyclic voltammograms, as described in **Equation** (**1**).^[^ ^30^, ^41^, ^42^ ^]^ The results are summarized in Table S2 (Supporting Information). After the introduction of SPTTPAB, the *q* values of all SPTTPAB/IrO_2_ samples were higher than IrO_2_ (56.95 mC cm^−2^), among which 5% SPTTPAB/IrO_2_ had the highest *q* value of 105.7 mC cm^−2^ (**Figure** **5**a; Figure S5, Supporting Information). Additionally, the number of active sites of the catalyst can also be estimated indirectly by the electrochemical active surface area (ECSA),^[^ ^43^, ^44^ ^]^ which is calculated by measuring the double‐layer capacitance (C_dl_) of the material (Table S2, Supporting Information).^[^ ^45^, ^46^ ^]^ The C_dl_ value of 5% SPTTPAB/IrO_2_ was calculated from the CV plot of Faraday intervals and was significantly higher than the other samples, reaching 47.72 mF cm^−2^ (Figure S6; Figure 5c, Supporting Information). This was mainly attributed to the incorporation of SPTTPAB, which significantly reduced the degree of IrO_2_ aggregation, thus amplifying the specific surface area of the catalyst and effectively increasing the number of active sites on the catalyst. However, when too much SPTTPAB was added, the catalytic activity decreased dramatically, which was mainly due to the organic chains blocking the active sites of the catalyst.^[^ ^47^ ^]^ Meanwhile, to better understand the differences in OER activity, the intrinsic catalytic activity of these prepared catalysts can be compared using their turnover frequency (TOF) values.^[^ ^11^ ^]^ Figure S7 (Supporting Information) present the calculated TOF of IrO_2_ and SPTTPAB/IrO_2_ at 1.9 V (vs RHE). It can be seen that the TOF values of SPTTPAB/IrO_2_ exhibit a trend of increasing first and then decreasing with the gradual increase SPTTPAB ratios. The 5% SPTTPAB/IrO_2_ obtain the largest TOF value (2.81 × 10^−4^ s^−1^), which is a 40.5% enhancement over pure IrO_2_ (Table S3, Supporting Information). The specific activity was acquired by normalizing the current to ECSA. The ECSA normalized results indicate that the intrinsic catalytic activity of IrO_2_ improved significantly after a small amount of SPTTPAB was coated on its surface (Figure S8, Supporting Information). These results indicate that the SPTTPAB coating has a significant impact on OER activity. On the other hand, comparing the CV curves of different samples in acid artificial seawater (Figure 5b), it can be found that the *q* value tends to be more linear with the change of SPTTPAB ratio (Table S2, Supporting Information). This is because the SPTTPAB coating improves the electrochemical active area of the catalyst, and enhances the dispersion and hydrophilicity of the material. Additionally, the coating layer provides additional anti‐impurity interference ability to the catalyst in acid artificial seawater, which makes the improvement of catalyst performance more obvious. The redox peaks near 0 V correspond to acid seawater erosion of the catalyst. It can be found that after the introduction of SPTTPAB, the redox peak is significantly inhibited, indicating that the intermediate layer can maintain the stability of the catalyst (Figure 5b). Moreover, two pairs of redox peaks near 0.9 and 1.2 V corresponding to Ir^3+^/Ir^4+^ and Ir^4+^/Ir^5+^ transitions were significantly enhanced after the integration of SPTTPAB, suggesting that the encapsulation of SPTTPAB promoted the electrochemical activity of the catalysts.^[^ ^48^ ^]^

We then used electrochemical impedance spectroscopy to assess the charge transfer capability at the catalyst interface.^[^ ^49^ ^]^ As shown in Figure 5d, the Nyquist plots exhibit decreasing semicircle diameters, indicating that the charge transfer impedance (Rct) of SPTTPAB/IrO_2_ is lower than that of IrO_2_,^[^ ^50^ ^]^ suggesting that the introduction of SPTTPAB enhances the interfacial charge transfer capacity of the catalyst. This can be interpreted as that the addition of a certain proportion of SPTTPAB conjugated polymer enhances the electrical conductivity of the SPTTPAB/IrO_2_ catalysts,^[^ ^51^ ^]^ while the ‐SO_3_H group grafted on the surface of SPTTPAB/IrO_2_ catalysts creates a rapid channel for the proton transport, which significantly reduces the proton transport resistance.^[^ ^52^ ^]^ In summary, the enhanced proton and electron conductivity of SPTTPAB/IrO_2_ and its multi‐layer porous structure are the reasons for its excellent OER catalytic performance.

In addition to the OER activity, the erosion resistance of the catalyst in seawater is indispensable. The corrosion induced by Cl^−^ ions in direct seawater electrolysis is a substantial challenge, resulting in the corrosion of the electrode surface through stress and pitting corrosion.^[^ ^53^ ^]^ We evaluated the corrosion resistance of IrO_2_ and SPTTPAB/IrO_2_ by corrosion experiments,^[^ ^54^, ^55^ ^]^ and the corresponding corrosion current and potential data are depicted in Figure S9 (Supporting Information). The SPTTPAB/IrO_2_ has a lower corrosion current and a higher corrosion potential than pure IrO_2_, revealing its strong corrosion resistance.^[^ ^56^, ^57^ ^]^ The erosion resistance of SPTTPAB/IrO_2_ may arise from the porous structure of the sample surface. Based on this hypothesis, we used the BET method to characterize SPTTPAB/IrO_2_ and IrO_2_. The nitrogen adsorption‐desorption isotherms for all samples exhibited standard IV curves and hysteresis loops (Figure S10, Supporting Information), indicating that these samples possess a favorable porous structure.^[^ ^58^ ^]^ The pore sizes of the IrO_2_ samples were primarily concentrated ≈1.6  and 3.2 nm, whereas the proportion of 1.6 nm pores in SPTTPAB/IrO_2_ was significantly increased after SPTTPAB coating (Figure 5e,f). The presence of these micropores makes it difficult for Cl^−^ to reach the surface of IrO_2_, and water molecules can readily pass through. In addition, the introduction of SPTTPAB coating also improves the specific surface area of the catalyst, among which 5% SPTTPAB/IrO_2_ has the largest specific surface area (130.2 m^2^ g^−1^), the number of micropores below 2 nm is up to 1.026 × 10^16^ g^−1^ (Table S4, Supporting Information).

Due to the high concentration of Cl^−^ ions in seawater electrolytes, these Cl^−^ ions can occupy a significant number of active sites on the catalyst. Furthermore, side reactions involving Cl^−^ ions during the seawater electrolysis take precedence over the oxygen evolution reaction. Therefore, under constant H⁺ ion concentration, the current density achieved by the catalyst in seawater electrolyte is generally lower than that obtained in H_2_SO_4_ electrolyte. As shown in Figure 5g, the current density of IrO_2_ between 1.44 and 1.62 V in acidic seawater electrolytes shows a noticeable increase compared to that in H_2_SO_4_ electrolytes. This suggests that Cl^−^ begins to participate in the reaction at this potential. In contrast, the side reaction of SPTTPAB/IrO_2_ in seawater electrolytes is obviously weakened, and the current density of SPTTPAB/IrO_2_ at high potential shows almost no attenuation compared with that in H_2_SO_4_ electrolyte, which indicated that SPTTPAB effectively blocked the Cl^−^, and enhanced the erosion resistance of SPTTPAB/IrO_2_. Furthermore, with an increase in SPTTPAB content, the ability of SPTTPAB/IrO_2_ to inhibit side reactions is improved (Figure 4c). As shown in Figure 5h, the inhibition mechanism could involve two key aspects. First, the structure of SPTTPAB features numerous micropores, allowing the passage of small water molecules while blocking the direct contact of larger Cl^−^ ions with IrO_2_.^[^ ^59^ ^]^ Second, the anionic ‐SO_3_H groups grafted onto SPTTPAB generate significant resistance to Cl^−^ in artificial seawater due to charge repulsion.^[^ ^52^ ^]^

**Figure 6:**
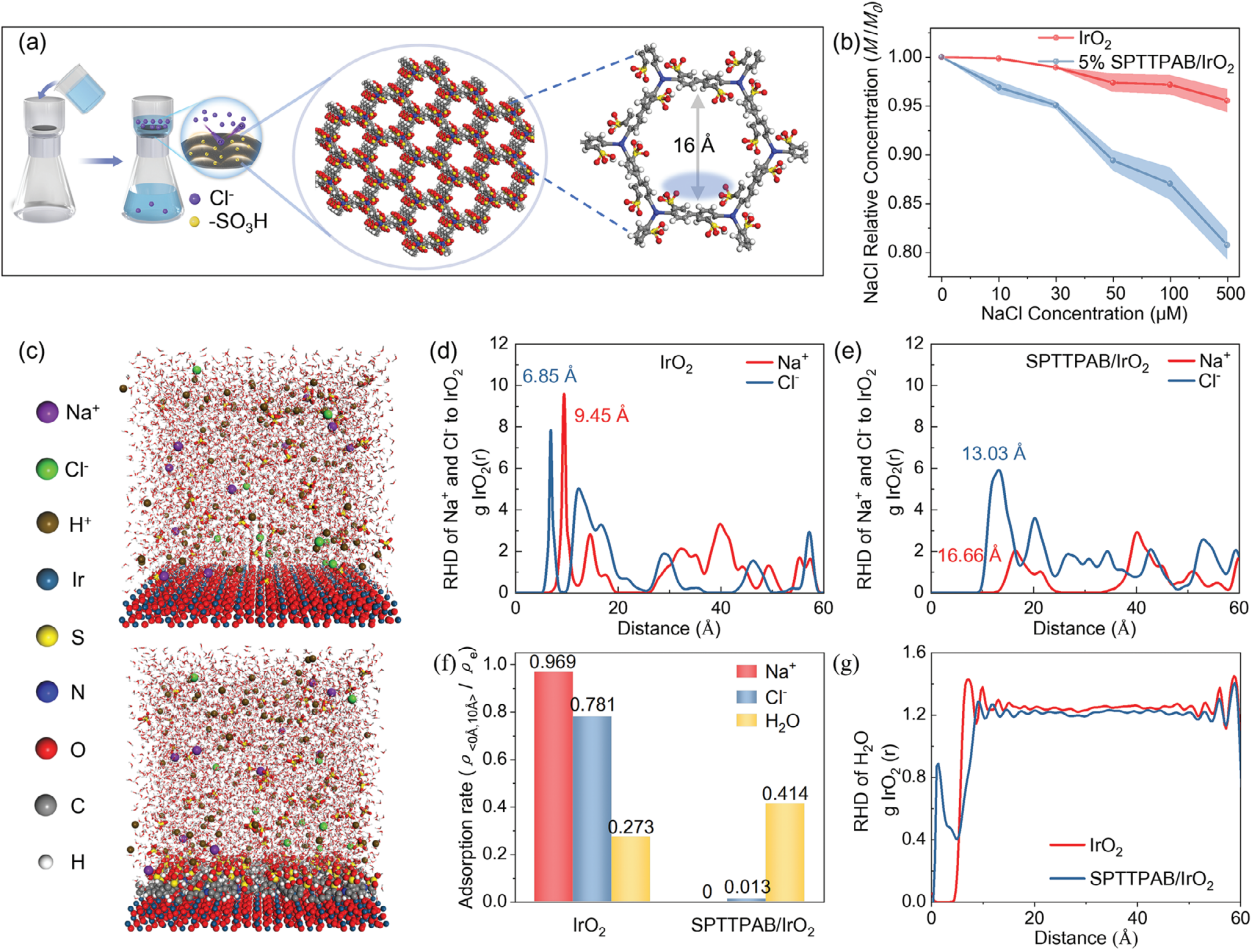
a) Diagram of the experimental device to verify the ion filtration capacity of the SPTTPAB and schematic of the structure of the SPTTPAB. b) Comparison of the filtration capacity of SPTTPAB/IrO_2_ and IrO_2_ for NaCl solutions with different concentrations. c) MD simulated the adsorption properties of Cl^−^, Na^+^, and H_2_O on IrO_2_ and SPTTPAB/IrO_2_ in seawater. The intensity of Na^+^ and Cl^−^ adsorbed on the d) IrO_2_ e) SPTTPAB/IrO_2_ in seawater. f) IrO_2_ and SPTTPAB/IrO_2_ absorption rates for each component in seawater. g) The intensity of H_2_O molecule adsorbed on the IrO_2_ and SPTTPAB/IrO_2_ in seawater.

To verify the Cl^−^ ions screening ability of SPTTPAB/IrO_2_, we designed the following experiments. As shown in **Figure** **6**a 5% SPTTPAB/IrO_2_ ink was coated on the filter paper, and a certain concentration of NaCl solution was slowly passed through the filter paper. The filtrate was collected and titrated with silver nitrate to determine the NaCl concentration. Results indicated that SPTTPAB/IrO_2_ coated filter paper plays a prominent role in intercepting NaCl, resulting in a marked decrease of NaCl in the filtrate. This effect intensified with increasing NaCl concentration, demonstrating that SPTTPAB/IrO_2_ exhibits substantial resistance to Cl^−^ ions (Figure 6b).

Further, we unveil the adsorption states of impurity ions on IrO_2_ and SPTTPAB/IrO_2_ surfaces by MD simulations (Figure 6c). Herein, the adsorption strength of ions on the catalyst surface was quantitatively analyzed by calculating the radial distribution function (RDF), which describes the relationship between strength and distance. RDF represents the strength of ions and catalysts at a specific distance *r*, calculated by Equation S1 (Supporting Information). As shown in Figures 6d and Figures 6e, for pristine IrO_2_, the first adsorption peak of Cl^−^ appeared at the 6.85 Å position, compared to the first Cl^−^ adsorption peak of SPTTPAB/IrO_2_ at 13.03 Å, suggesting that SPTTPAB can effectively prevent Cl^−^ from coming into direct contact with the internal IrO_2_. Similarly, the comparison of Na^+^ distribution peaks shows that SPTTPAB also blocks Na^+^. Comparing the average concentration of each component in the range of 10 Å, it can be seen that SPTTPAB/IrO_2_ shows a strong ion filtration ability, and barely any Na^+^ and Cl^−^ can reach the catalyst surface through the SPTTPAB organic polymer framework (Figure 6f). Moreover, by comparing the locations of the H_2_O molecule distribution peaks of IrO_2_ and SPTTPAB/IrO_2_, it can be concluded that the ‐SO_3_H on the surface of SPTTPAB also enhances the hydrophilicity of the catalyst to a certain extent (Figure 6g).

Summarizing the above discussions, the excellent OER performance of SPTTPAB/IrO_2_ catalyst is attributed to the following factors: (1) SPTTPAB increases the specific surface area of IrO_2_, thereby exposing more active sites. (2) SPTTPAB enhances the electron and proton conductivity of the system. (3) The porous structure of SPTTPAB facilitates selective screening of substances that reach the catalyst surface, effectively limiting the involvement of other anions from the electrolyte in the reaction and thus improving catalytic selectivity.

## 3 Conclusion

In summary, based on porous conductive organic compounds synthesized from polyaniline, an anodic seawater electrolysis catalyst with a specific resistance to ion erosion was designed. The catalyst with the ability of proton/electron conduction presents a sulfonate group concentration of 5.62 × 10^−4^ mol g^−1^, with micropore below 2 nm numbering 1.026 × 10^16^ g^−1^. This unique structure selectively screens substances that reach the catalyst surface, blocking the adsorption of 98.62% of Cl^−^ ions on the catalyst and inhibiting coupling reactions between metal atoms and chlorine, thereby enhancing catalytic selectivity and activity in seawater electrolysis. As a result, the most cost‐effective 5 wt.% SPTTPAB/IrO_2_ shows the overpotential was only 283 mV@10 mA cm^−2^, with a Tafel slope as low as 16.33 mV dec^−1^, which reduced 13.8% and 37.8% compared to commercial IrO_2_, respectively. Moreover, SPTTPAB/IrO_2_ exhibited outstanding performance in integrated seawater electrolysis tests, with a 35.3% improvement over commercial IrO_2_ to 69 mA cm^−2^@1.9 V, and a degradation rate (0.018 mA h^−1^) that is only 24.6% of that of commercial IrO_2_. This strategy offers a practical pathway for designing seawater electrolysis electrocatalysts with enhanced performance and durability.

## 4 Experimental Section

### Materials

1,3,5‐Tribromobenzene (99%), 4‐(diphenylamino)phenylboronic acid (98%), Pd(PPh_3_)_2_Cl_2_ were purchased from Beijing Yinokai Technology Co., LTD. Anhydrous ferric chloride, sulfuric acid, sodium chloride, calcium chloride, magnesium chloride, petroleum ether, dichloroethane (99.9%), and anhydrous ethanol purchased from Shanghai Aladdin Biochemical Technology Co., LTD. Iridium oxide (IrO_2_, 98%, Ir 85%) was purchased from Shanxi Kaida Chemical Co., LTD. The Nafion solution (D520, EW 1100) was acquired from Chemours Chemicals Co., Ltd.

### Synthesis of TTPAB

1,3,5‐tris(4‐diphenylamino‐phenyl)benzene (TTPAB) was prepared by Suzuki coupling method, and the specific synthesis process is shown in Figure 1. 1,3,5‐tribromobenzene (0.35 g), diphenylaminobenzoborate (1.32 g), Pd(PPh_3_)_2_Cl_2_ (30 mg) and NaOH (0.24 g) was dissolved in toluene (20 mL), and then heated at 110 °C under nitrogen atmosphere for 20 min. After cooling to room temperature, the mixture was extracted with saturated salt water and dried with anhydrous MgSO_4_. The TTPAB was separated into white solid powder by column chromatography with a yield of 58%. The NMR spectrum of TTPAB is presented in Figure S1 (Supporting Information).

### Synthesis of SPTTPAB/IrO_2_

TTPAB was synthesized by an oxidative coupling reaction catalyzed by FeCl_3_, referring to previous work.^[^ ^60^, ^61^, ^62^, ^63^ ^]^ TTPAB (1, 3 wt%, 5, and 10 wt.%), IrO_2_ (100 mg), and FeCl_3_ (20 mg) were dispersed in dichloroethane and stirred overnight under nitrogen at room temperature. Upon completion of polymerization, the mixture was precipitated in methanol, followed by filtration and repeated washing with methanol and water to remove FeCl_3_. The resulting PTTPAB/IrO_2_ product was vacuum‐dried at 80 °C for 24 h. Subsequently, PTTPAB/IrO_2_ powder (0.1 g) was dispersed in 2 mL H_2_SO_4_ and stirred mechanically at 55 °C for 3 h. The reaction mixture was quenched by dropping into ice‐cold ethanol, and the precipitate was washed with ethanol until the pH reached 6–7 to remove residual acid. The final SPTTPAB/IrO_2_ product was dried under vacuum at 80 °C for 24 h. The surface morphology of SPTTPAB is shown in Figure S2 (Supporting Information).

### Structural Characterization

Fourier transform infrared spectroscopy (FT‐IR) was performed using a Nicolet 6700 spectrometer (Thermo Fisher) with KBr pellets. X‐ray diffraction (XRD, D8 Advance) was used to analyze the crystal structure of the samples. Transmission electron microscopy (TEM, IEM‐2100F) was employed to examine the morphology, chemical composition, and elemental distribution. The particle size and Zeta potential of the samples were analyzed by dynamic light scattering (DLS, Malvern, Zetasizer Nano ZSE). The specific surface area and pore distribution were determined via the Brunauer‐Emmett‐Teller (BET) method. Elemental composition and chemical states were analyzed by X‐ray photoelectron spectroscopy (XPS, ESCALAB 250Xi) under Mg‐Kα (1253.6 eV) radiation.

### Electrochemical Characterization

The electrochemical properties of the SPTTPAB/IrO_2_ catalyst were evaluated at room temperature in 0.1 m HClO_4_ using a three‐electrode system. A glassy carbon (GC) electrode (5 mm diameter) served as the working electrode, with platinum foil as the counter electrode and a saturated calomel electrode (SCE) as the reference electrode. All potentials were calibrated against a reversible hydrogen electrode (RHE) (E(RHE) = E(SCE) + 0.3 V). Catalyst ink was prepared by dispersing 2 mg of the catalyst in a mixture of 320 µL isopropyl alcohol, 80 µL deionized water, and 8 µL D520. An electrode with a catalyst loading of 0.2 mg cm^−2^ was fabricated. Cyclic voltammetry (CV) was performed at a scan rate of 20 mV s^−1^ within a potential window of 0 to 1.2 V. The voltammetric charge (*q*) was measured by integral cyclic voltammetry, as shown in Equation (1).

(1) $$q=\int_{E1}^{E2}\frac{\left|i\right|}{\upsilon }dE$$

where *i* is the current density, *v* is the scanning rate of 20 mV s^−1^, and *E* is the scanning potential between 0 and 1.2 V versus RHE.

Linear sweep voltammetry (LSV) for the oxygen evolution reaction was conducted at 5 mV s^−1^. Electrochemical impedance spectroscopy (EIS) was measured over a frequency range of 0.1 Hz to 10 kHz at 1.57 V, with a sinusoidal perturbation amplitude of 10 mV. The double‐layer capacitance was evaluated at different scan rates between 1 and 1.1 V. Prior to all tests, the electrodes were conditioned by cycling between 1 and 1.4 V at 50 mV s^−^¹ for 40 cycles. All electrochemical measurements were carried out using a CHI 660 electrochemical workstation.

### Integral Water Decomposition Test

Integral water decomposition tests were carried out in a two‐electrode system using different mass fractions of SPTTPAB/IrO_2_ as the anode catalyst and commercial 50% Pt/C as the cathode catalyst. For comparison, commercial IrO_2_ was used as the reference for the anode catalyst. Titanium felt was used as the anode substrate and carbon paper as the cathode substrate, and the catalyst was coated on the surface to make an electrode sheet with an active area of 1 × 1 cm^2^. The final loading capacities of anode and cathode catalysts were 1.5 ± 0.2 and 0.2 ± 0.05 mg cm^−2^, respectively. Two electrolytes were prepared: a 0.5 m H_2_SO_4_ solution and an acid seawater solution consisting of five times diluted artificial seawater mixed with 0.5 m H_2_SO_4_. CV activation experiments were carried out in the range of 0 ≈1.9 V voltage window at a scanning rate of 50 mV s^−1^. LSV experiments were carried out at a voltage window of 1.2 to 1.9 V at a scanning rate of 5 mV s^−1^. All electrochemical tests were also performed at the CHI 660 electrochemical workstation.

## Conflict of Interest

The authors declare no conflict of interest.

## Supporting information



Supporting Information

## Data Availability

The data that support the findings of this study are available from the corresponding author upon reasonable request.
