# Supplementary material for: High Catalytic Selectivity of Electron/Proton Dual‐Conductive Sulfonated Polyaniline Micropore Encased IrO2 Electrocatalyst by Screening Effect for Oxygen Evolution of Seawater Electrolysis
Source: Adv Sci (Weinh). 2024 Dec 4;12(4):2412862. doi: 10.1002/advs.202412862 (PMC11775546; doi:10.1002/advs.202412862)
Supplement: Supplementary file 1 — Supporting Information [file ADVS-12-2412862-s001.docx]

**High Catalytic Selectivity of Electron/Proton Dual-Conductive Sulfonated Polyaniline Micropore Encased IrO_2_ Electrocatalyst by Screening Effect for Oxygen Evolution of Seawater Electrolysis**

*Yuhan Shen, Shengqiu Zhao*, Fanglin Wu, Hao Zhang, Liyan Zhu, Mingjuan Wu, Tian Tian, and Haolin Tang**

Y. Shen, S. Zhao, F. Wu, H. Zhao, L. Zhu, M. Wu, T. Tian, H. Tang

State Key Laboratory of Advanced Technology for Materials Synthesis and Processing, Wuhan University of Technology, Wuhan, 430070, P. R. China

E-mail: zhaoshengqiu@whut.edu.cn; thln@whut.edu.cn

S. Zhao, H. Tang

National energy key laboratory for new hydrogen-ammonia energy technologies, Foshan Xianhu Laboratory, Foshan, 528200, P. R. China

H. Tang

Hubei Key Laboratory of Fuel Cell, Wuhan, 430070, P. R. China

**Supplementary Material**

**1. Computational section**

**2. SPTTPAB/IrO_2_ analysis**

**Figure S1.** Nuclear magnetic resonance spectroscopy of TTPAB.

**Figure S2.** SEM image of SPTTPAB.

**Figure S3.** Particle size, zeta potential, and electronic conductivity of 5% IrO_2_ and SPTTPAB/IrO_2_.

**Figure S4.** TEM image and XRD pattern of SPTTPAB/IrO_2_ after OER.

**Figure S5.** CV integrated electric charge (*q*) of IrO_2_ and SPTTPAB/ IrO_2_.

**Figure S6.** Cyclic voltammetry curves were recorded at different scanning rates of IrO_2_ and SPTTPAB/IrO_2_.

**Figure S7.** TOF of IrO_2_ and SPTTPAB/IrO_2_.

**Figure S8.** ECSA normalized LSV curves.

**Figure S9.** Corrosion potential and corrosion current density.

**Figure S10.** N_2_ adsorption-desorption isotherm of IrO_2_ and SPTTPAB/IrO_2_

**Table S1.** Elemental mass distribution of 5% SPTTPAB/IrO_2_.

**Table S2.** Electrochemical properties of IrO_2_ and SPTTPAB/IrO_2_.

**Table S3.** Detail TOF of IrO_2_ and SPTTPAB/IrO_2_.

**Table S4.** The physical properties IrO_2_ and SPTTPAB/ IrO_2_.

Computational section

Molecular dynamic (MD) simulations were applied to investigate the solution component diffusion on IrO_2_(1 1 0) and SPTTPAB/IrO_2_(1 1 0) surfaces. The solid-liquid interface calculations for our considered electrolyte on IrO_2_(1 1 0) and SPTTPAB/IrO_2_(1 1 0) surface were conducted with Universal force field (UFF)^[1]^ using the Forcite module in Materials Studio (MS) 2020^[2]^. The dimensional lengths of the IrO_2_(1 1 0) slab are 58.68 Å × 49.96 Å in the xoy plane. The electrolyte contains 50 H_2_SO_4_, 10 NaCl, and 5467 H_2_O molecules in a rectangular box with length scales of 58.68 Å × 49.96 Å× 57.79 Å. After geometry optimization, the aqueous solution was placed on the optimized IrO_2_(1 1 0) or SPTTPAB/IrO_2_(1 1 0 surface. All MD calculations were performed under the NVT ensemble (T=298.0 K) with a time step of 1 fs and a total simulation time of 400 ps, during which simulation trajectories were recorded every 4000 steps. The temperature was controlled by a Nose-Hoover thermostat. The Ewald scheme and atom-based cutoff method (i.e., a radius of 12.5 Å) were applied to treat electrostatic and van der Waals (vdW) interactions, respectively. The partial charges for each atom are assigned by the (charge balance equation) QEq method. The density profiles of Na^+^, Cl^-^, SO_4_^2-^, H^+^, and H_2_O along perpendicular directions were analyzed. The solid-liquid interface snapshots were also displayed. RDF represents the intensity of substance A and substance B at a specific distance r, which was calculated using the equation:

$g（r）=\frac{V}{N_{A}N_{B}}\left\langle\sum_{i=1}^{N_{A}} \frac{N_{\mathrm{iB}}(r，\Delta r)}{4\pi r^{2}\Delta r} \right\rangle$ (S1)

Where, *V* is the total volume of the simulated system, and *N_A_* and *N_B_* are the number of particles A and B, *N_iB_* (*r*, Δ*r*) is the number of B particles in the range *r* to Δ*r*.


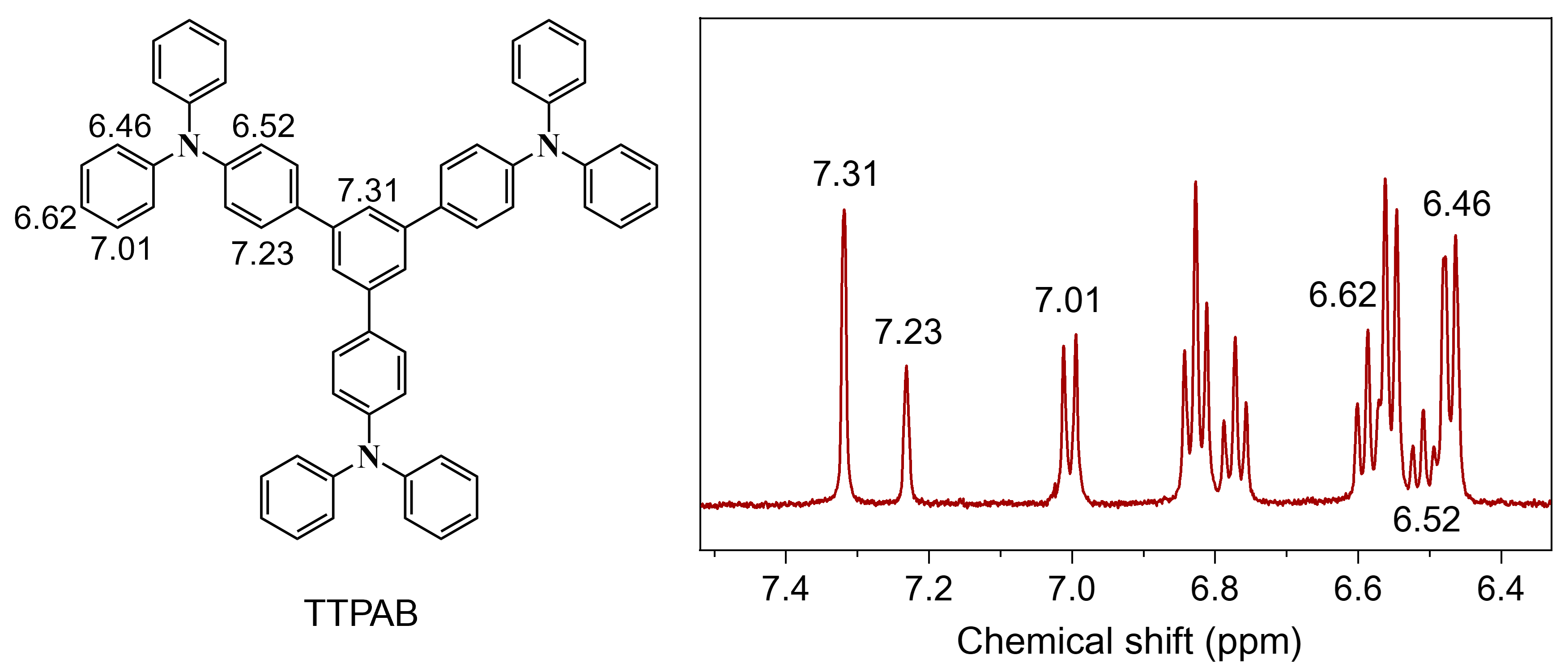


**Figure S1.** Nuclear magnetic resonance spectroscopy of TTPAB.


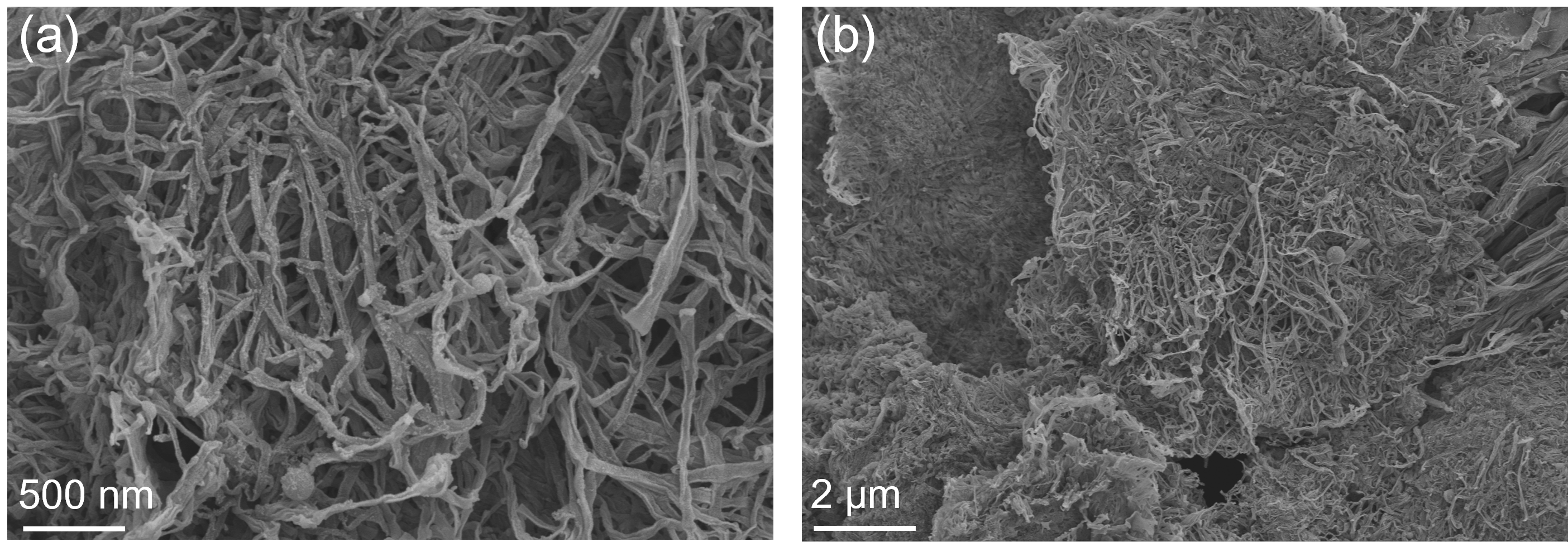


**Figure S2.** SEM image of SPTTPAB.


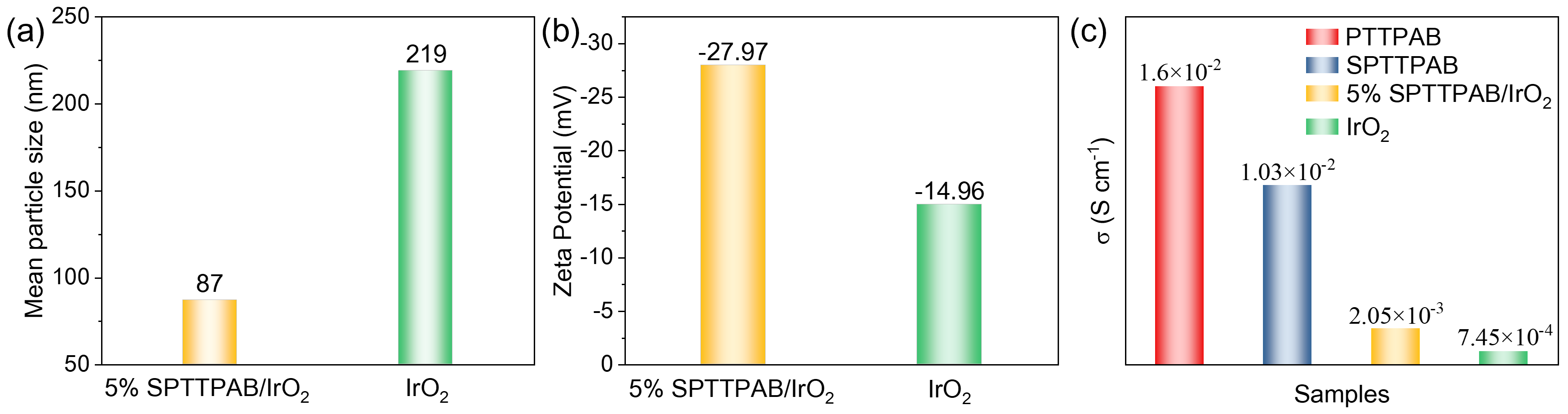


**Figure S3.** (a) particle size, (b) zeta potential, (c) electronic conductivity of 5% SPTTPAB/IrO_2_ and IrO_2_.


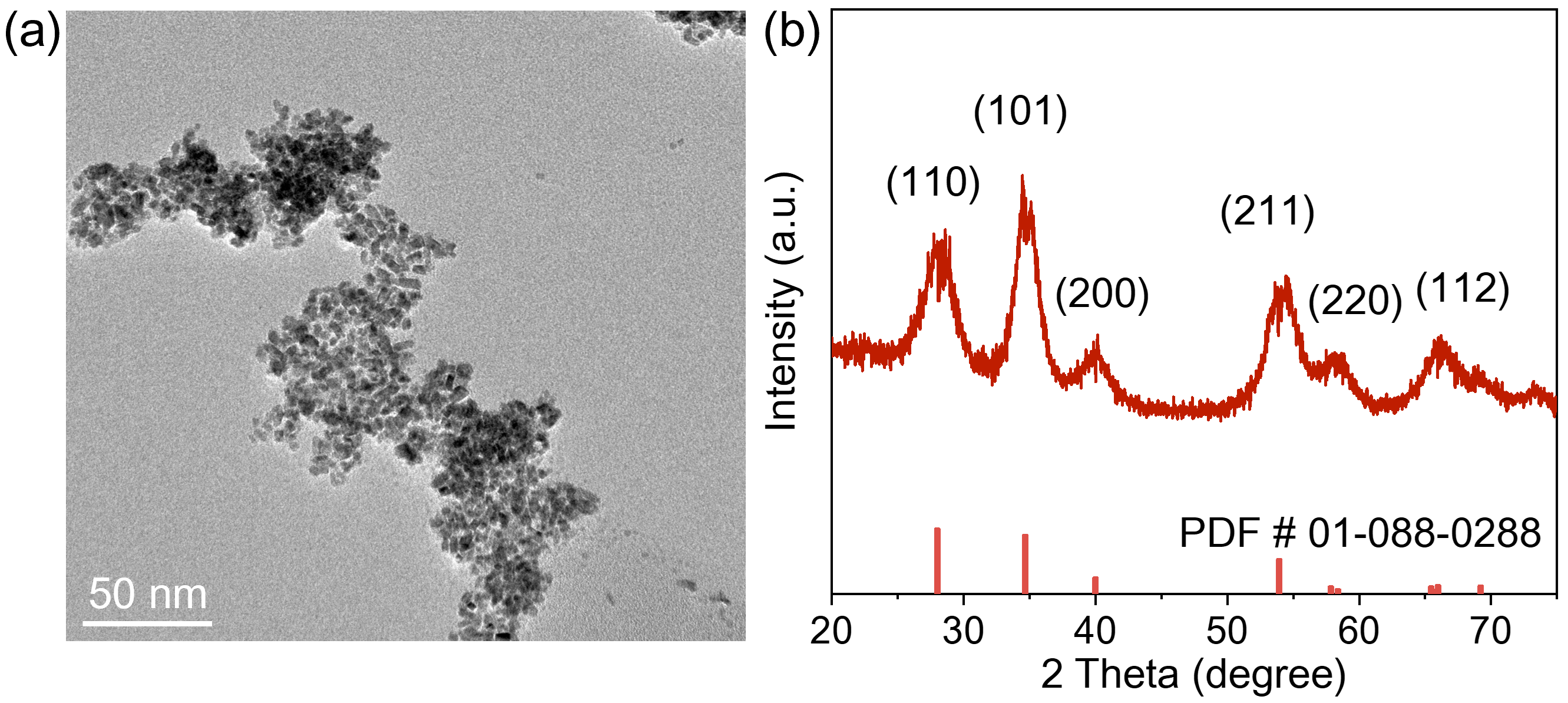


**Figure S4.** (a) TEM image and (b) XRD pattern of SPTTPAB/IrO_2_ after OER.


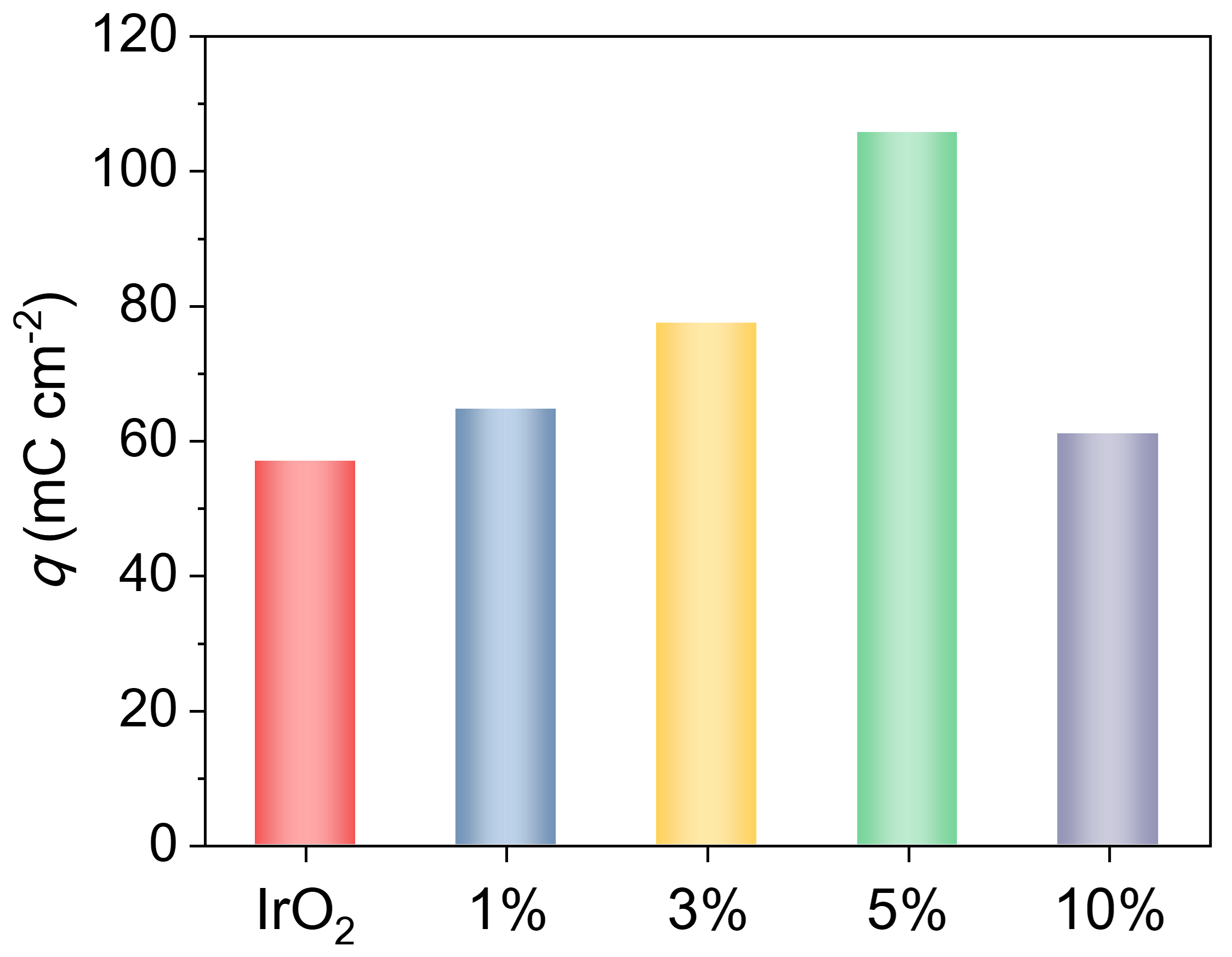


**Figure S5.** CV integrated electric charge (*q*) of SPTTPAB/ IrO_2_ and IrO_2_ in H_2_SO_4_ electrolyte.


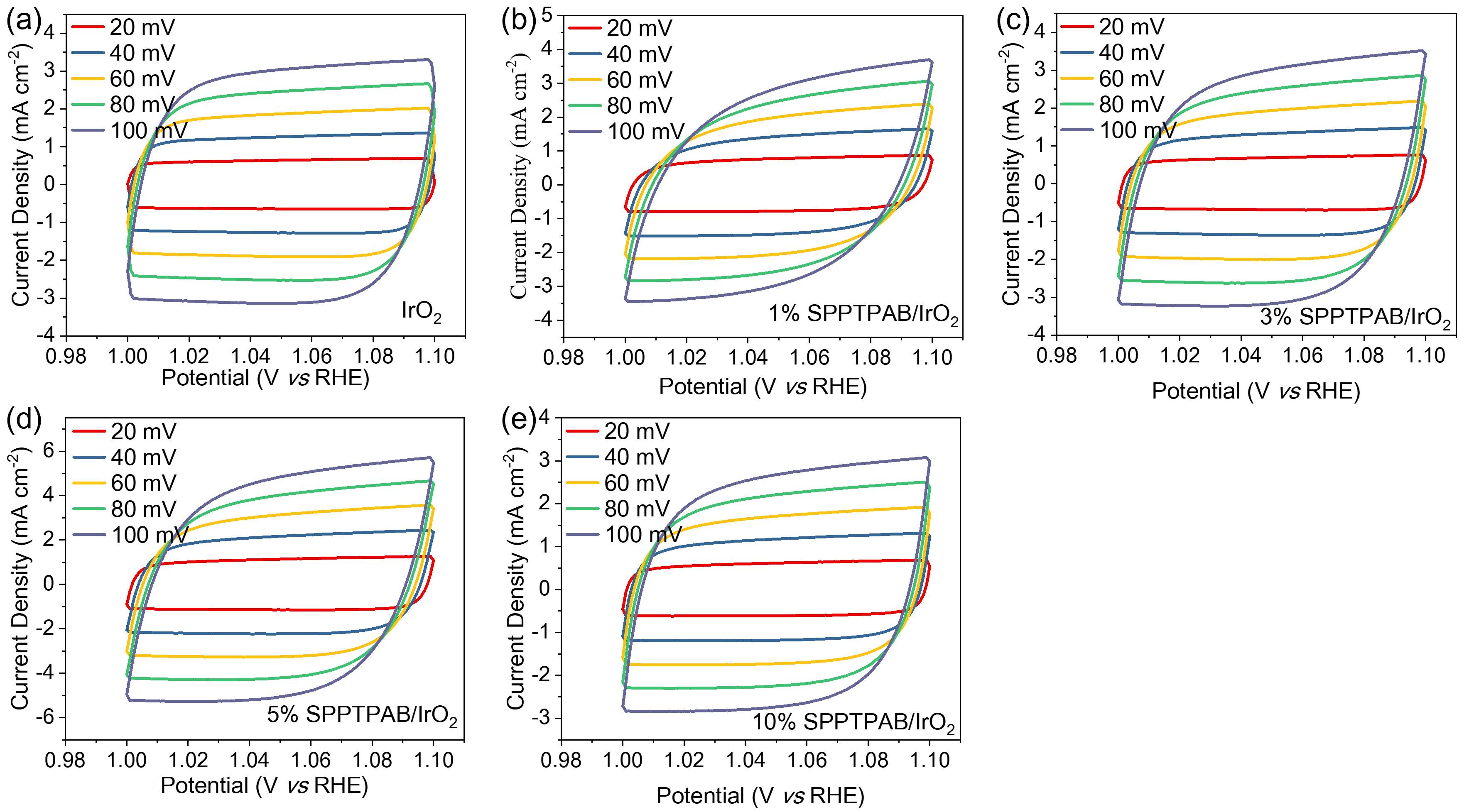


**Figure S6.** Cyclic voltammetry curves recorded at different scanning rates of (a) IrO_2_, (b) 1% SPTTPAB/IrO_2_, (c) 3% SPTTPAB/IrO_2_, (d) 5% SPTTPAB/IrO_2_ and (e)10% SPTTPAB/IrO_2_.


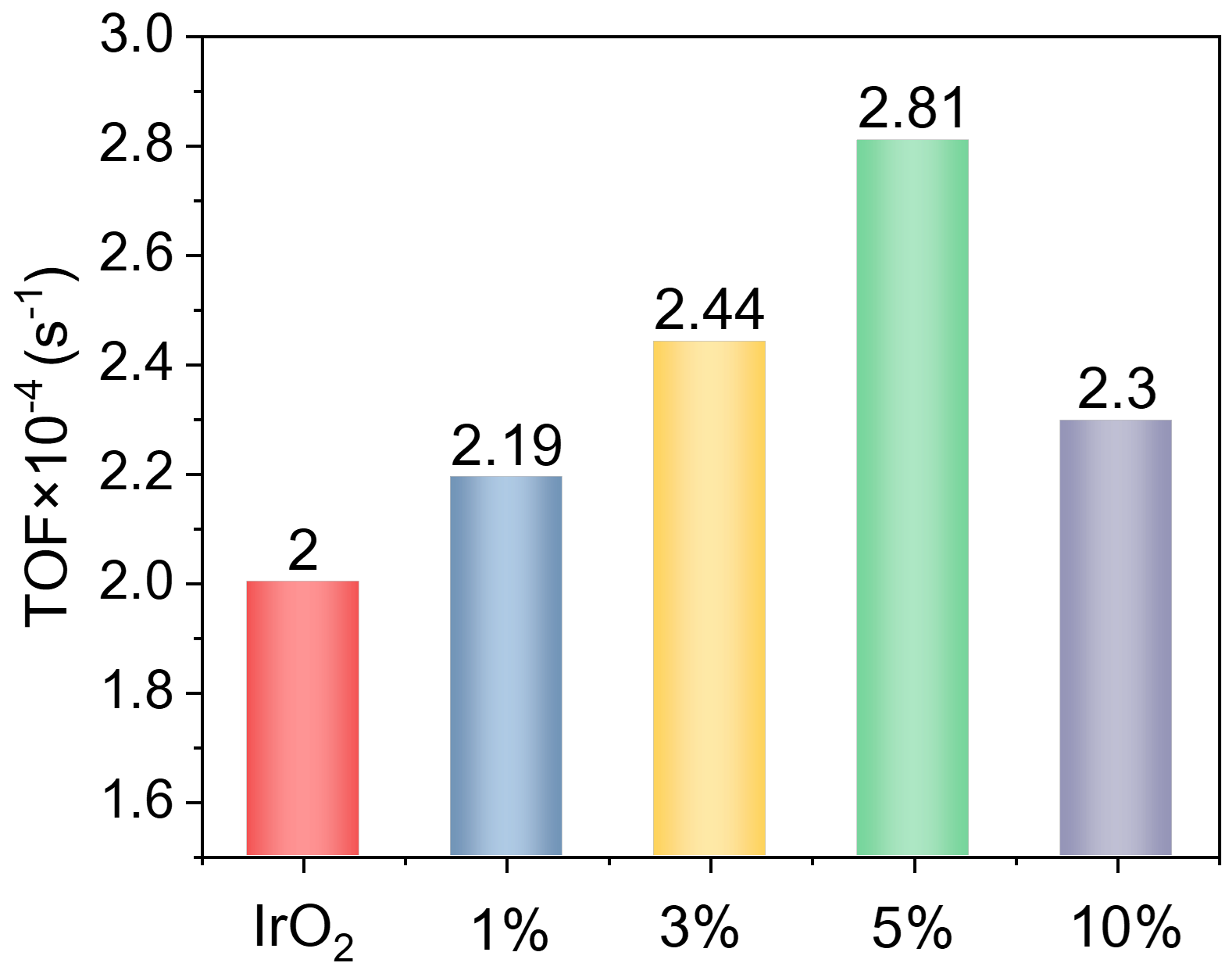


**Figure S7.** TOF of IrO_2_ and SPTTPAB/IrO_2_ at 1.9 V (vs RHE) in acid seawater electrolyte.


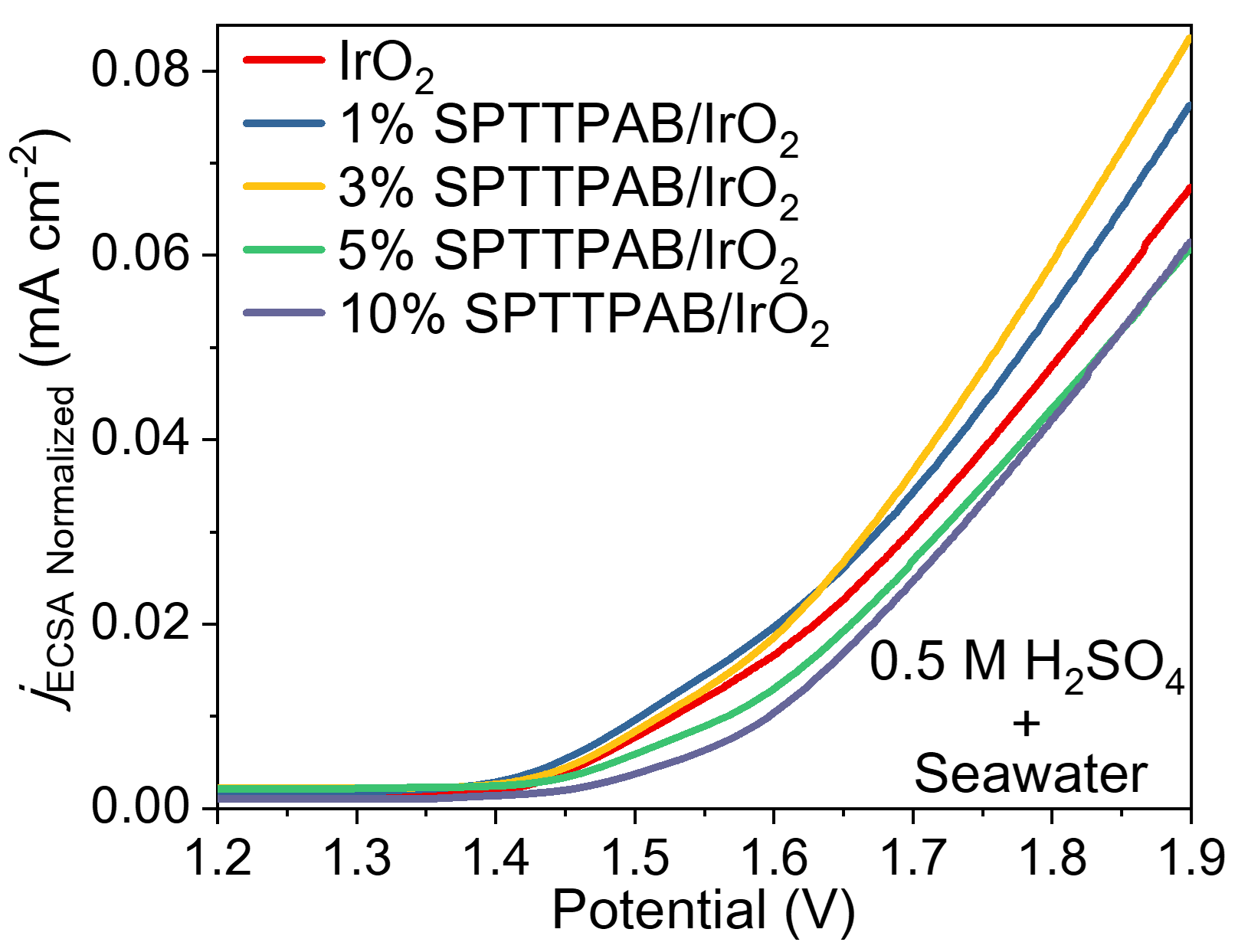


**Figure S8.** The ECSA normalized LSV curves of IrO_2_ and SPTTPAB/IrO_2_ in acid seawater electrolyte.


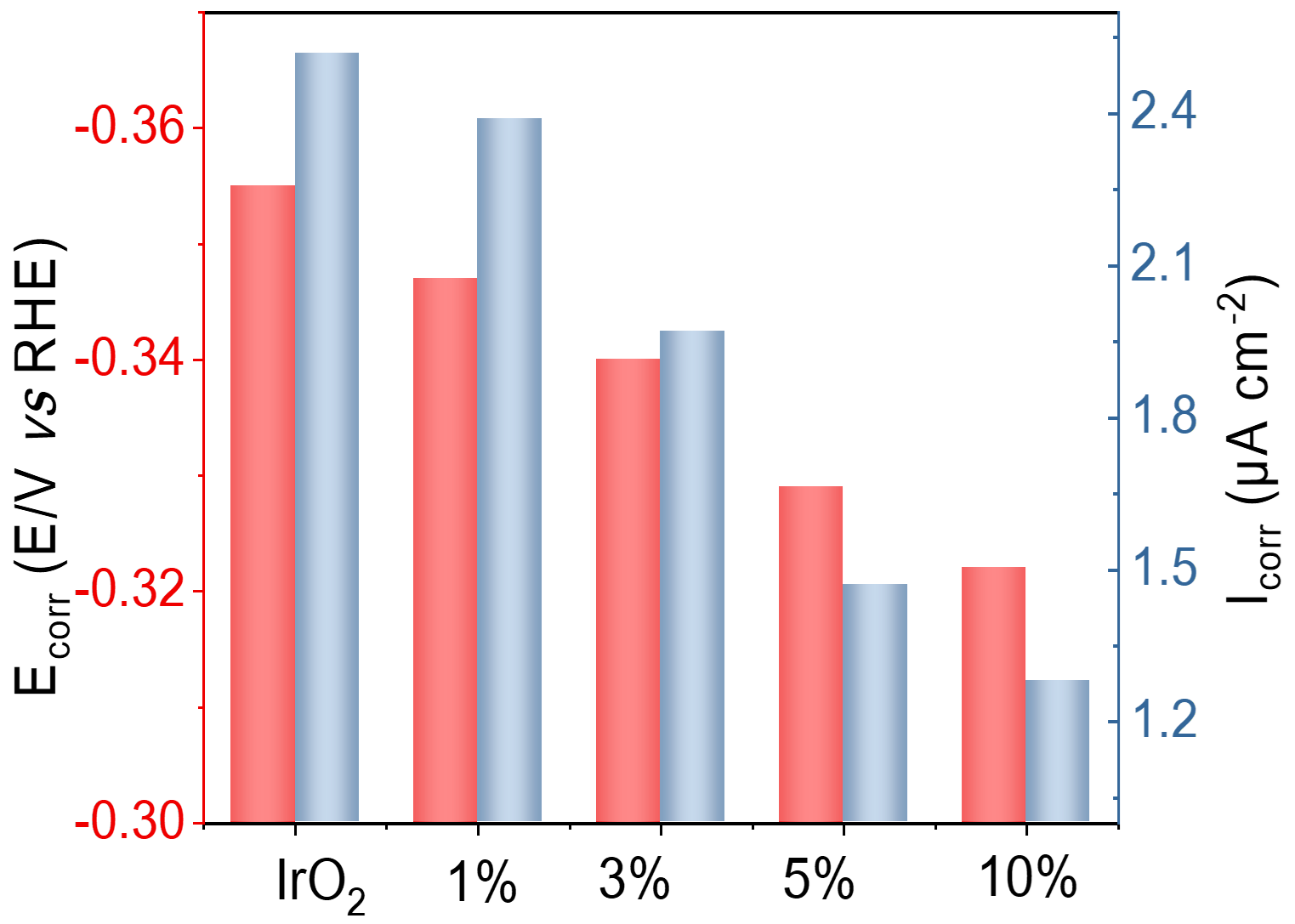


**Figure S9.** Corrosion potential and corrosion current density of IrO_2_ and SPTTPAB/IrO_2_ in acid seawater electrolyte.


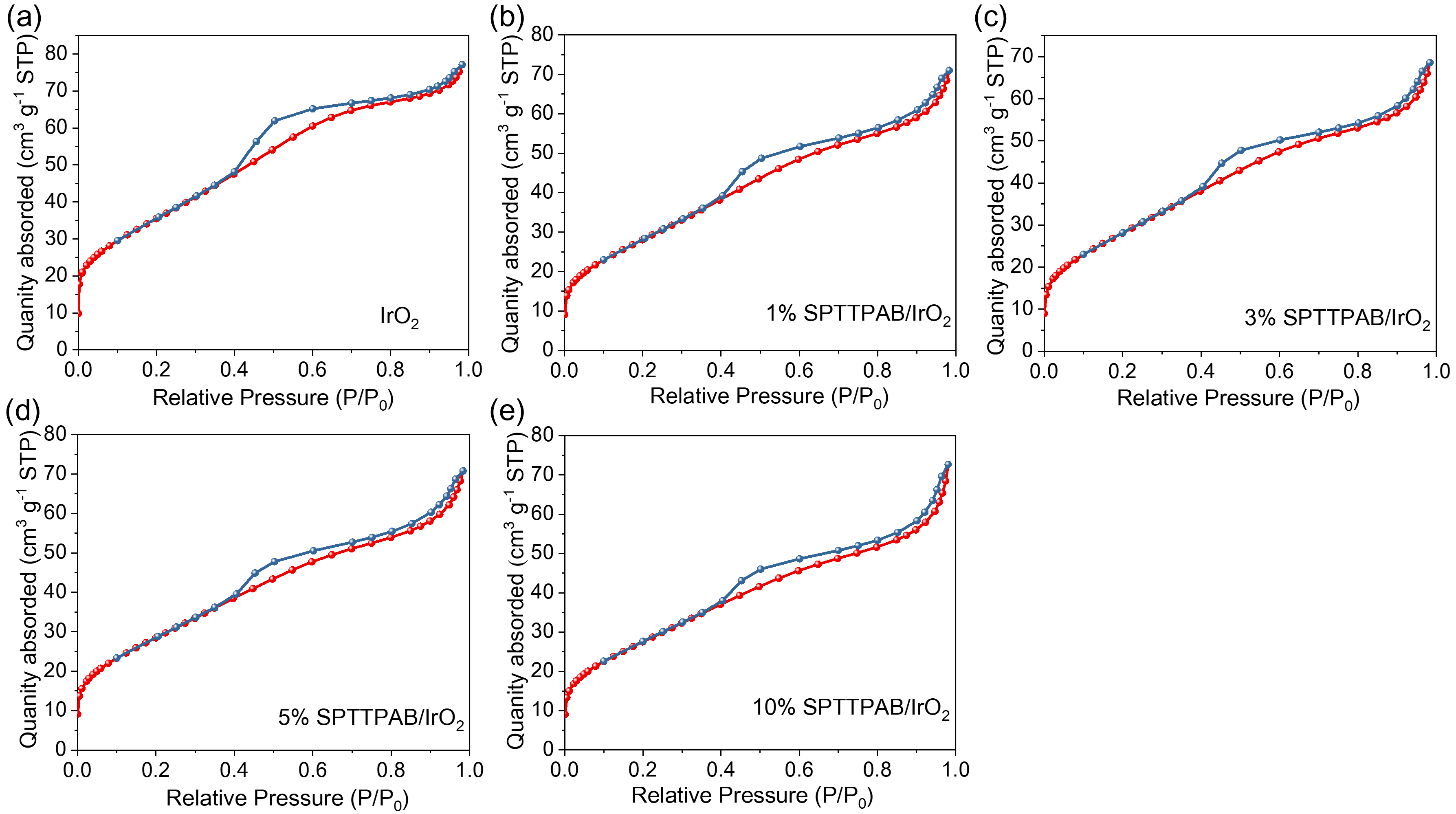


**Figure S10.** N_2_ adsorption-desorption isotherm of (a) IrO_2_, (b) 1% SPTTPAB/IrO_2_, (c) 3% SPTTPAB/IrO_2_, (d) 5% SPTTPAB/IrO_2_, and (e)10% SPTTPAB/IrO_2_.

**Table S1.** Elemental mass distribution of 5% SPTTPAB/IrO_2_.

| Elements | Mass (%) |
| --- | --- |
| S 2p | 1.8 |
| N 1s | 0.4 |
| C 1s | 6.6 |
| O 1s | 21.6 |
| Ir 4f | 69.5 |

**Table S2.** Electrochemical properties. (i) IrO_2_, (ii) 1% SPTTPAB/IrO_2_, (iii) 3% SPTTPAB/IrO_2_, (iv) 5% SPTTPAB/IrO_2_ and (Ⅴ)10% SPTTPAB/IrO_2_.

| Sample | *q*  (mC cm^-2^)  In H_2_SO_4_ | *q*  (mC cm^-2^)  In acid seawater | C_dl_  (mF cm^-2^) | ECSA | Tafel slope  (mV dec^-1^) |
| --- | --- | --- | --- | --- | --- |
| (i) IrO_2_ | 56.95 | 61.75 | 30.69 | 767.25 | 44.01 |
| (ii) 1% SPTTPAB/IrO_2_ | 64.65 | 72.85 | 29.69 | 742.25 | 39.26 |
| (iii) 3% SPTTPAB/IrO_2_ | 77.4 | 97.35 | 30.16 | 754 | 34.45 |
| (iv) 5% SPTTPAB/IrO_2_ | 105.7 | 111.85 | 47.72 | 1193 | 30.38 |
| (Ⅴ) 10% SPTTPAB/IrO_2_ | 61 | 94.7 | 38.6 | 965 | 34.71 |

**Table S3.** TOF of (i) IrO_2_, (ii) 1% SPTTPAB/IrO_2_, (iii) 3% SPTTPAB/IrO_2_, (iv) 5% SPTTPAB/IrO_2_ and (Ⅴ)10% SPTTPAB/IrO_2_.

| Sample | TOF×10^-4^ (s^-1^) |
| --- | --- |
| (i) IrO_2_ | 2 |
| (ii) 1% SPTTPAB/IrO_2_ | 2.19 |
| (iii) 3% SPTTPAB/IrO_2_ | 2.44 |
| (iv) 5% SPTTPAB/IrO_2_ | 2.81 |
| (Ⅴ) 10% SPTTPAB/IrO_2_ | 2.3 |

**Table S4.** The physical property of synthesized. (i) IrO_2_, (ii) 1% SPTTPAB/IrO_2_, (iii) 3% SPTTPAB/IrO_2_, (iv) 5% SPTTPAB/IrO_2_ and (Ⅴ)10% SPTTPAB/IrO_2_.

| Sample | BET surface area  (m^2^ g^-1^) | Pore volume  (cm^3^ g^-1^) | Average Pore Diameter  (nm) | Pore volume  below 2 nm (cm^3^ g^-1^) |
| --- | --- | --- | --- | --- |
| (i) IrO_2_ | 102.5 | 0.11 | 4.4 | 0.0132 |
| (ii) 1% SPTTPAB/IrO_2_ | 104.9 | 0.11 | 3.92 | 0.0132 |
| (iii) 3% SPTTPAB/IrO_2_ | 104.9 | 0.11 | 4.06 | 0.014 |
| (iv) 5% SPTTPAB/IrO_2_ | 130.2 | 0.12 | 3.68 | 0.022 |
| (Ⅴ) 10% SPTTPAB/IrO_2_ | 106.2 | 0.11 | 4.14 | 0.014 |

*Note*：The average diameter of micropores with a diameter below 2 nm is 1.6 nm, and

according to formula: $N=\frac{V_{total (\leq2 nm)}}{V_{1.6 nm}}$, the number of micropores below 2 nm for 5% SPTTPAB/IrO_2_ can be calculated to be 1.026 × 10^16^ g^-1^.

References

[1] Rappé, A. K.; Casewit, C. J.; Colwell, K. S.; Goddard III, W. A.; Skiff, W. M. UFF, a full periodic table force field for molecular mechanics and molecular dynamics simulations. *J. Am. Chem. Soc.* **1992**, *114*, 10024-10035.

[2] BIOVIA, D. S. Materials Studio, 9.0. Dassault Systemes, San Diego, **2020**.
